# Methicillin resistance and the biofilm phenotype in *Staphylococcus aureus*

**DOI:** 10.3389/fcimb.2015.00001

**Published:** 2015-01-28

**Authors:** Hannah McCarthy, Justine K. Rudkin, Nikki S. Black, Laura Gallagher, Eoghan O'Neill, James P. O'Gara

**Affiliations:** ^1^Department of Microbiology, School of Natural Sciences, National University of IrelandGalway, Ireland; ^2^Department of Clinical Microbiology, Royal College of Surgeons in IrelandDublin, Ireland

**Keywords:** biofilm, LPXTG proteins, Atl, PIA, *mecA*, methicillin resistance, c-di-AMP, *Staphylococcus aureus*

## Abstract

Antibiotic resistance and biofilm-forming capacity contribute to the success of *Staphylococcus aureus* as a human pathogen in both healthcare and community settings. These virulence factors do not function independently of each other and the biofilm phenotype expressed by clinical isolates of *S. aureus* is influenced by acquisition of the methicillin resistance gene *mecA*. Methicillin-sensitive *S. aureus* (MSSA) strains commonly produce an *icaADBC* operon-encoded polysaccharide intercellular adhesin (PIA)-dependent biofilm. In contrast, the release of extracellular DNA (eDNA) and cell surface expression of a number of sortase-anchored proteins, and the major autolysin have been implicated in the biofilm phenotype of methicillin-resistant *S. aureus* (MRSA) isolates. Expression of high level methicillin resistance in a laboratory MSSA strain resulted in (i) repression of PIA-mediated biofilm production, (ii) down-regulation of the accessory gene regulator (Agr) system, and (iii) attenuation of virulence in murine sepsis and device infection models. Here we review the mechanisms of MSSA and MRSA biofilm production and the relationships between antibiotic resistance, biofilm and virulence gene regulation in *S. aureus*.

## Biofilm formation by staphylococci and its medical significance

Implantable medical devices have revolutionized modern healthcare; however attachment to these devices by surface adhering bacteria resulting in biofilm formation and device related infections (DRIs) substantially impact patient morbidity and mortality. Biofilms formed by staphylococci have for many decades been recognized as the most frequent cause of biofilm-associated infections with *Staphylococcus epidermidis* and *S. aureus* being among the most common etiologic agents of DRIs (Davies, 2003; Otto, 2013). Infections associated with biofilms are difficult to treat because the biofilm matrix and phenotypic characteristics of the bacteria confer resistance to the host immune response and the action of antimicrobial drugs (O'Gara and Humphreys, 2001).

All implanted medical devices are susceptible to colonization by staphylococci and staphylococcal biofilm-associated infections have been associated with devices ranging from implanted catheters to prosthetic heart valves, cardiac pacemakers, contact lenses, cerebrospinal fluid shunts, joint replacements and intravascular lines (Donlan and Costerton, 2002; Hall-Stoodley et al., 2004). Damaged host tissue is also a risk factor for developing biofilm-associated infection. *S. epidermidis* and *S. aureus*, which are part of the normal skin flora are opportunistic pathogens that can cause deep-seated skin infections for example in burn or post-operative wounds (Hammond et al., 2011). They are also known to form biofilms on damaged heart valves, leading to the development of life-threatening infective endocarditis (Claret et al., 2007; Nethercott et al., 2013).

*S. epidermidis*, unlike the more virulent *S. aureus*, relies almost exclusively on its ability to colonize and form biofilms on implanted medical devices to cause clinical infections (O'Gara and Humphreys, 2001; Christner et al., 2012) and a strong correlation has been reported between the pathogenicity of *S. epidermidis* and its ability to colonize implanted medical devices (Vuong and Otto, 2002). The ability of *S. aureus* to form biofilms on implanted medical devices or damaged host tissue is also a key virulence factor for this pathogen, especially in healthcare settings where antibiotic usage is high and such biofilm formation represents a survival mechanism for the bacteria (Høiby et al., 2010). Among the most studied mechanism of biofilm formation is the production of the *icaADBC* operon-encoded polysaccharide intercellular adhesion (PIA) or poly-N-acetylglucosamine (PNAG) by both *S. aureus* and *S. epidermidis* (O'Gara, 2007; Joo and Otto, 2012). However, biofilm formation independent of the *ica* operon has also been described in both *S. epidermidis* and *S. aureus* (Hussain et al., 1997; Fitzpatrick et al., 2005; Tormo et al., 2005; Qin et al., 2007; O'Neill et al., 2008; Schroeder et al., 2009; Shahrooei et al., 2009; Geoghegan et al., 2010).

In this review, we describe different mechanisms used by methicillin-sensitive *S. aureus* (MSSA) and methicillin-resistant *S. aureus* (MRSA) isolates to form biofilm and how the acquisition or loss of methicillin resistance impacts on, not only the biofilm phenotype of *S. aureus*, but also on global gene regulation and virulence.

## Biofilm formation by methicillin-sensitive *S. aureus*

The first described mechanism of *S. aureus* biofilm formation involved PIA/PNAG production (Cramton et al., 1999, 2001) that had earlier been described in *S. epidermidis* (Mack et al., 1994; Heilmann et al., 1996a,b; Heilmann and Götz, 1998). PIA is a glycan of β-1,6-linked 2-acetamido-2-deoxy-D-glucopyranosyl residues with a net positive charge that promotes intercellular aggregation and attachment of cells to inert surfaces (Rohde et al., 2010). The *ica* operon consists of four biosynthesis genes, *icaA, icaD, icaB*, and *icaC* and a divergently transcribed repressor, *icaR* (Conlon et al., 2002; Götz, 2002). The majority of research on the activity of the *ica* locus has been conducted in *S. epidermidis* but there is high nucleotide sequence homology between the *ica* loci of *S. epidermidis* and *S. aureus* and 78% identity at the amino acid level (Cramton et al., 1999). An early study demonstrated that a transposon mutation in the *ica* operon of *S. epidermidis* impaired biofilm formation and PIA production (Heilmann et al., 1996b). Subsequent studies showed that *S. aureus* also commonly uses *icaADBC*-encoded PIA as a mechanism of biofilm formation (Cramton et al., 1999, 2001).

Carriage of the *ica* locus is a characteristic of most clinical *S. aureus* strains (Cramton et al., 1999; Fowler et al., 2001; Rohde et al., 2001). One of the first reports of PNAG-independent *S. aureus* biofilm production was described in bovine mastitis isolates, which formed biofilm mediated by the biofilm associated protein (Bap) (Cucarella et al., 2001, 2004). An early study with the human *S. aureus* isolate UAMS-1, also revealed an *icaADBC*-independent biofilm phenotype under *in vitro* and *in vivo* conditions (Beenken et al., 2004).

Investigations into PIA-dependent and PIA-independent mechanisms of biofilm formation identified a correlation between methicillin susceptibility and biofilm in *S. aureus* (Fitzpatrick et al., 2005; O'Neill et al., 2007, 2008; Houston et al., 2011). This relationship between the biofilm phenotype and β-lactam susceptibility was first investigated in *S. epidermidis* by Mempel et al. who reported that variable levels of PIA production were significantly associated with different levels of β-lactam susceptibility in phenotypic variants (Mempel et al., 1994, 1995). The significant association between methicillin susceptibility in *S. aureus* and *ica*-dependent biofilm formation was first reported when PIA production was found to be essential for biofilm formation by MSSA but not MRSA (O'Neill et al., 2007). MSSA biofilms are significantly induced in growth media supplemented with NaCl, which is known to activate *ica* operon expression (Fitzpatrick et al., 2006; O'Neill et al., 2007). Furthermore, MSSA biofilms are susceptible to sodium metaperiodate treatment (which oxidizes polysaccharide bonds) and are resistant to treatment with proteinase K (O'Neill et al., 2007). Deletion of the *ica* locus abolished biofilm-forming capacity among clinical MSSA isolates that are amenable to genetic manipulation, as did mutation of the staphylococcal accessory regulator *sarA* (Valle et al., 2003; O'Neill et al., 2007).

Building on an original finding that four clinical MRSA isolates were capable of producing *icaADBC*-independent biofilm (Fitzpatrick et al., 2005), our laboratory reported differences in the environmental regulation of biofilm formation among 32 *S. aureus* isolates from intensive care units (15 MSSA and 17 MRSA strains) and found that NaCl-induced, PIA-dependent biofilm was more likely to be associated with MSSA biofilm formation (Fitzpatrick et al., 2006). A follow-up study of a large collection of 212 *S. aureus* isolates from device-related infections (114 MRSA and 98 MSSA) representing five clonal complexes (CC5, CC8, CC22, CC30, and CC45) further demonstrated that MSSA strains were more likely to produce NaCl-induced, PIA-mediated biofilm whereas MRSA biofilm was induced in media supplemented with glucose and was PIA-independent (O'Neill et al., 2007).

## Biofilm formation by MRSA strains

The *ica* locus was found to be redundant for MRSA biofilm formation (O'Neill et al., 2007). Unlike the NaCl-induced biofilm expressed by MSSA clinical isolates, biofilm formation by MRSA isolates was significantly more likely to be induced by the addition of glucose to the growth medium, which is associated with the acidification of the culture media (O'Neill et al., 2007). MRSA biofilms are resistant to sodium metaperiodate treatment but are susceptible to proteinase K treatment, implicating protein adhesins in this biofilm phenotype (O'Neill et al., 2007).

As observed in MSSA isolates, mutation in *sarA* also abolished biofilm formation by clinical MRSA isolates (Beenken et al., 2004; O'Neill et al., 2007). SarA is a known repressor of four major extracellular proteases, namely SspA, SspB, Aur, and ScpA (Karlsson et al., 2001) and it is proposed that impaired glucose-induced biofilm formation by MRSA *sarA* mutants may be associated with upregulation of protease activity, which can inhibit *S. aureus* biofilm production (Marti et al., 2010).

A deletion in the accessory gene regulator (*agr*) system enhanced biofilm formation by clinical MRSA strains but had no significant effect on biofilms formed by MSSA strains (O'Neill et al., 2007). In contrast to *sarA, agr* positively regulates expression of the four major proteases (Shaw et al., 2004). Additionally, mutations in the *agr*-regulated Aur metalloprotease and the SplABCDEF serine proteases increased biofilm formation and reduced detachment from established biofilms (Boles and Horswill, 2008). Similarly, the serine protease inhibitor phenylmethylsulfonyl fluoride (PMSF) enhanced biofilm formation, further implicating increased protease activity as the mechanism of *agr*-mediated dispersal from biofilms (Boles and Horswill, 2008). The positive impact of an *agr* mutation on MRSA biofilm formation is largely consistent with other research showing that deletion of *agr* can enhance *S. aureus* biofilm formation (Vuong et al., 2000; Beenken et al., 2003), whilst a separate study demonstrated that *agr* deletion does not significantly alter *icaADBC* expression or PIA production (Vuong et al., 2003). Interestingly, the reduced pH of cultures grown in media supplemented with glucose, was associated with repression of the *agr* system (Regassa et al., 1992). Furthermore, activation of the *agr* system was shown to initiate biofilm dispersal (Boles and Horswill, 2008). The *agr* locus encodes two divergent transcripts, RNAII and RNAIII driven by the P2 and P3 promoters, respectively (Novick et al., 1993). RNAII comprises four genes, *agrBDCA*, that encode for the synthesis of secreted autoinducing peptide AIP. At threshold concentrations, AIP binds to the sensor histidine kinase AgrC, which phosphorylates AgrA and activates the P2 and P3 promoters, resulting in upregulated *agr* expression and increased transcription of the RNAIII effector molecule. Exogenous addition of AIP to biofilms induced dispersal from established biofilms by upregulating the *agr* system and subsequently increasing protease production (Boles and Horswill, 2008). The enhanced biofilm phenotype observed in *agr* mutants is also attributed to the loss of production of the RNAIII-encoded delta-toxin, which has surfactant properties (Vuong et al., 2000, 2004). Biofilm detachment by *agr*^+^ strains is proposed to require the formation of a film of amphipathic delta-toxin molecules expressed at the biofilm/fluid interface which can inhibit the hydrophobic interactions between bacterial cell surfaces, thus lowering surface tension and causing cell detachment from the biofilm matrix (Vuong et al., 2004).

### Role of LPXTG-anchored surface proteins in MRSA biofilm formation

*S. aureus* expresses 28 surface proteins, 21 of which are predicted to contain LPXTG binding motifs required for normal display on the cell surface (Ton-That et al., 2000; Mazmanian et al., 2001). Anchoring of these LPXTG motif-containing proteins to peptidoglycan is catalyzed by Sortase, an extracellular transpeptidase encoded by the *srtA* gene (Mazmanian et al., 2000). A *srtA* mutation affected biofilm accumulation by clinical MRSA strains from three clonal complexes and had no impact on *ica*-dependent biofilms formed by MSSA strains (O'Neill et al., 2008). As noted earlier Bap was the first LPXTG-anchored surface protein implicated in *ica*-independent *S. aureus* biofilm formation but is rarely found in human isolates (Cucarella et al., 2001; Tormo et al., 2005).

The fibronectin-binding proteins (FnBPs) were subsequently shown to play an important role in *ica*-independent biofilm formation by human MRSA isolates (O'Neill et al., 2008; Shanks et al., 2008; Vergara-Irigaray et al., 2009). The FnBPs are multi-functional surface proteins with an N-terminal A domain and a C-terminal wall spanning LPXTG-anchoring domain separated by tandem repeats involved in binding to fibronectin (Schwarz-Linek et al., 2003; Meenan et al., 2007). Both FnBP proteins, FnBPA and FnBPB, are involved in the accumulation phase of MRSA biofilm formation in hospital and community isolates under static and flow conditions, with each individual protein capable of complementing the biofilm defect of a double *fnbpAB* mutant (O'Neill et al., 2008; McCourt et al., 2014). The FnBPs do not contribute to PIA-mediated biofilm formation by clinical MSSA strains (O'Neill et al., 2008). Expression of the FnBPs was constitutive in a MRSA isolate producing an FnBP-dependent biofilm but restricted to the exponential growth phase in MSSA isolates that form PIA biofilms (Geoghegan et al., 2013). The N-terminal of the FnBPA protein, specifically residues 166–498 comprising the N2 and N3 subdomains of the A domain, has been implicated in the biofilm phenotype via a Zn(2+)-dependent mechanism (O'Neill et al., 2008; Geoghegan et al., 2013). A separate study from the Lasa laboratory, demonstrated that MRSA strain 132 was capable of producing either acid stress-induced FnBP-dependent biofilm or osmotic stress-induced exopolysaccharide-mediated biofilm (Vergara-Irigaray et al., 2009). Furthermore, the 132 *fnbpAB* mutant displayed significantly impaired colonization and biofilm formation compared to the isogenic 132 *ica* mutant in a murine model of subcutaneous catheter-related infection (Vergara-Irigaray et al., 2009). Recently the formation of bacterial biofilm-like aggregates in human synovial fluid by MRSA and MSSA strains was attributed to expression of the FnBPs as well as the fibrinogen binding proteins ClfA and ClfB, identifying biofilm and in particular LPXTG-wall anchored proteins as important virulence determinants in staphylococcal joint infections (Dastgheyb et al., 2014).

In MRSA strains where the FnBPs have not (yet) been implicated in protein dependent biofilm, a number of other LPXTG proteins may be important. The accumulation associated protein (Aap), which is 54% identical to the SasG protein of *S. aureus* (Corrigan et al., 2007) was first identified in *S. epidermidis* and is known to play a well-defined role in the biofilm phenotype (Hussain et al., 1997; Rohde et al., 2005; Conrady et al., 2008, 2013; Conlon et al., 2014; Schaeffer et al., 2015). SasG, which is found in approximately 50% of human clinical isolates, has also been implicated in the intercellular accumulation phase of *ica*-independent biofilm formation (Corrigan et al., 2007; Sung et al., 2008). SasG promotes intercellular aggregation via homo-oligomerization of the SasG protein (Kuroda et al., 2008) with biofilm formation promoted by the repeated B domains of the SasG protein (Geoghegan et al., 2010). The B repeat regions promote *ica*-independent biofilm formation in a Zn(2+)-dependent manner through the formation of extended fibrils on the cell surface (Geoghegan et al., 2010; Gruszka et al., 2012). Protein A also promotes polysaccharide-independent biofilm but does not need to be anchored to the cell wall via its LPXTG motif to do so, with exogenous Protein A capable of promoting biofilm accumulation (Merino et al., 2009). However, the role or otherwise of SasG and protein A to staphylococcal biofilm-associated infections has not yet been investigated. Readers are referred to a companion review in this series focusing on staphylococcal proteinaceous biofilms by Pietro Speziale.

### Role of the major autolysin and extracellular DNA in MRSA biofilm formation

The major autolysin, Atl, which is involved in daughter cell separation, cell wall homeostasis and peptidoglycan turnover (Yamada et al., 1996; Biswas et al., 2006) plays a role in the early stages of *ica*-independent biofilm formation by clinical MRSA strains (Houston et al., 2011). This bifunctional peptidoglycan hydrolase was first implicated in the primary attachment of *S. epidermidis* to polystyrene (Heilmann et al., 1997; Biswas et al., 2006), with *atlE* mutants exhibiting significantly attenuated virulence in a rat central venous catheter infection model (Rupp et al., 2001). AtlE of *S. epidermidis* also possesses vitronectin-binding activity implicated in AtlE-mediated biofilm formation on plasma protein-coated polymer surfaces (Heilmann et al., 1997). Clinical MRSA strains that produce *ica*-independent biofilms require Atl for primary attachment to polystyrene surfaces (Houston et al., 2011).

The major autolysin of *S. aureus* is expressed as a 137.5 kDa pro-protein which is proteolytically processed to yield a 3.1 kDa signal sequence, a 17.6 kDa propeptide and two enzymatically active regions, a 63.3 kDa *N*-acetylmuramyl-L-alanine amidase enzyme and a 53.6 kDa endo-β-*N*-acetylglucosaminidase enzyme (Oshida et al., 1995). Catalytic activity of the amidase region is required for PIA-independent proteinaceous biofilm formation by hospital acquired MRSA (HA-MRSA) strains (Houston et al., 2011). The protease inhibitor PMSF, which increases the release of extracellular autolytic enzymes (Fournier and Hooper, 2000), and polyanethole sodium sulfanate, which inhibits autolytic activity without impairing growth (Wecke et al., 1986; Yabu and Kaneda, 1995), both prevented biofilm formation by HA-MRSA strains indicating a vital role for both the unprocessed Atl protein and the active amidase and glucosaminidase enzymes in early MRSA biofilm formation (Houston et al., 2011).

Construction of enzymatically inactive point mutations within the active regions of Atl revealed Atl-mediated cell lysis and the release of extracellular DNA (eDNA) by both the amidase and glucosaminidase regions as the mechanisms of Atl-mediated biofilm formation (Bose et al., 2012). Several studies have reported a role for eDNA in the *ica*-independent healthcare associated and community acquired MRSA (CA-MRSA) biofilm phenotypes (Izano et al., 2008; Lauderdale et al., 2010; Houston et al., 2011). In *S. aureus*, addition of DNase I to the culture media inhibited PIA-independent biofilm formation by HA-MRSA but did not significantly disperse mature MRSA biofilms implicating eDNA in the attachment and/or early stage of biofilm development (Houston et al., 2011). In the CA-MRSA strain USA300, the secreted thermostable nuclease enzyme, Nuc has also been shown to negatively impact on biofilm formation (Kiedrowski et al., 2011). In a mouse model of catheter infections, however, mutations in *nuc* and a second nuclease gene *nuc2* of the UAMS-1 MSSA strain were associated with reduced biofilm (Beenken et al., 2012) indicating that additional studies with MSSA and MRSA isolates, are needed to fully elucidate the role of these enzymes and extracellular nucleic acid in the biofilm phenotype *in vivo*.

**Figure 1:**
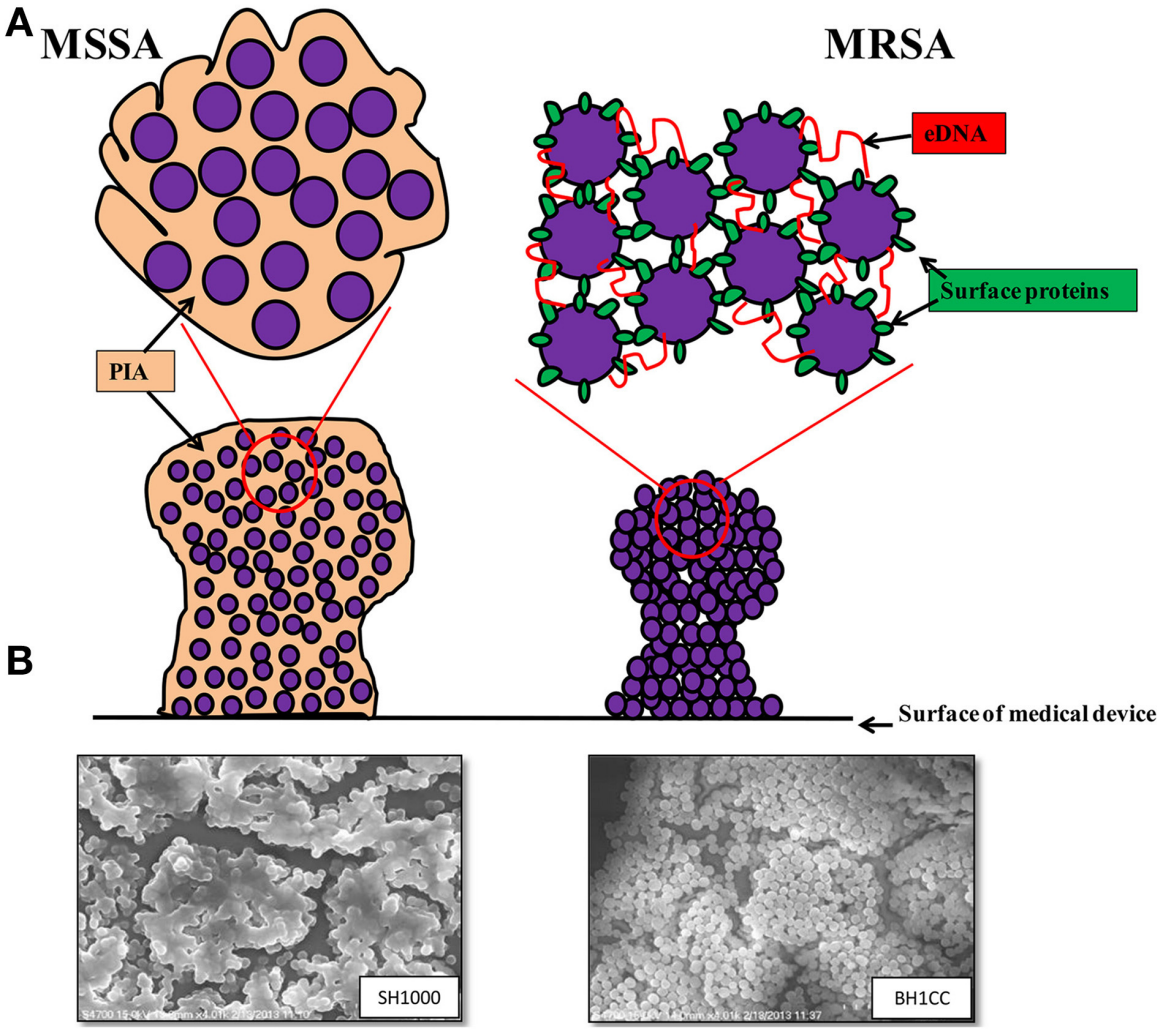
**(A)** Graphic representations of the biofilm phenotypes expressed by MSSA and MRSA. MSSA strains form *ica*-dependent, PIA-mediated biofilms whereas MRSA strains form biofilms independent of PIA and require surface proteins such as the fibronectin binding proteins, Atl-mediated cell lysis and eDNA for colonization of surfaces and biofilm accumulation. **(B)** Scanning electron micrographs (3500 × magnification) of biofilms formed by MSSA strain SH1000 grown in BHI media supplemented with 4% NaCl (left) and MRSA strain BH1CC grown in BHI supplemented with 1% glucose (right).

A study of *ica*-independent mechanisms of biofilm formation by *S. aureus* revealed that altered levels of autolysis were associated with defective biofilm production (Boles et al., 2010). Recently cytoplasmic proteins released during the stationary phase of growth, which may be a consequence of autolysis, have been shown to be part of the biofilm matrix of *S. aureus* HG003 (Foulston et al., 2014). Interestingly the release of cytoplasmic proteins during stationary phase appears to be in response to decreasing pH, which can be triggered by the addition of excess glucose to the growth media (Foulston et al., 2014) and it is worth noting that these same growth conditions promote Atl/FnBP-mediated MRSA biofilm formation (O'Neill et al., 2008). A simplified model of MSSA and MRSA biofilm mechanisms together with scanning electron micrographs of SH1000 (MSSA) and BH1CC (MRSA) biofilms is presented in Figure 1.

## How does methicillin resistance affect the biofilm phenotype and virulence?

### Methicillin resistance and biofilm

The mechanism(s) underpinning the association between the MRSA and MSSA biofilm phenotypes and utilization of polysaccharide and protein adhesins, respectively remains unclear. However, the level of resistance to beta-lactam antibiotics appears to be important for the biofilm phenotype. For instance, excision of the SCC*mec* element from the hospital MRSA strain BH1CC was associated with reduced FnBP-mediated biofilm forming capacity, presumably due to up-regulated protease activity (Pozzi et al., 2012; Rudkin et al., 2012). The relationship between methicillin resistance and biofilm is further complicated by the observation that MRSA strains can express either low level heterogeneous resistance (HeR) to methicillin or high-level, homogeneous resistance (HoR) (Keaton et al., 2013; Mwangi et al., 2013). Carriage of the *mecA* gene alone does not confer the HoR phenotype and additional genetic events are also needed (Pozzi et al., 2012; Mwangi et al., 2013; Dordel et al., 2014). In the laboratory isolation of homogeneously resistant strains from HeR strains is readily achieved by plating cell suspensions on media supplemented with high concentrations of oxacillin, the clinically used derivative of methicillin (100 μg/ml) (Pozzi et al., 2012; Dordel et al., 2014). In clinical isolates of MRSA the *ica* locus is present and expressed but PIA does not appear to be produced (O'Neill et al., 2007). In an engineered derivative of the laboratory strain 8325-4 carrying the *mecA* gene on a plasmid and expressing HoR, the *ica* operon was repressed >300-fold compared to its MSSA parent and was associated with a switch from PIA-dependent to proteinaceous biofilm (Pozzi et al., 2012). In contrast expression of HeR in 8325-4 did not significantly alter the biofilm phenotype (Pozzi et al., 2012). Extracellular protease activity was reduced in the 8325-4 HoR strain and 8235-4 HoR biofilms were dispersed with proteinase K implicating a protein adhesin in this phenotype (Pozzi et al., 2012). However, because this biofilm phenotype was independent of PIA/PNAG, the major autolysin or any of the LPXTG cell wall anchored proteins whereas clinical MRSA isolates express an FnBP/eDNA dependent biofilm, it appears that expression of homogeneous methicillin resistance is associated with at least two distinct biofilm phenotypes. It remains to be determined whether PBP2a expression exerts direct or indirect (e.g., via altered cell wall architecture) effects on biofilm production. In a murine device infection model 8325-4 and 8325-4 HoR were recovered in similar numbers from implanted catheters, suggesting that PIA/PNAG and protein adhesin-mediated biofilms may be equally effective for device colonization *in vivo* (Pozzi et al., 2012).

### Oxacillin resistance and virulence

High level PBP2a expression in MRSA is associated with repression of the Agr quorum sensing operon (Pozzi et al., 2012; Rudkin et al., 2012). Agr repression in MRSA is directly linked to *mecA* expression and the subsequent changes in cell wall architecture are accompanied by reduced cytotoxin production and attenuated virulence. The repression of Agr blocks the co-ordinated switch-on of toxin and enzyme secretion during the latter stages of bacterial growth *in vitro*, leaving the cell arrested in the surface protein expression stage of the Agr regulatory cycle (Rudkin et al., 2012). As noted above, increased surface protein expression and repression of extracellular protease production are also consistent with the protein-mediated MRSA biofilm phenotype (Pozzi et al., 2012).

Murine infection model studies showed that mice infected with the 8325-4 HoR strain were more likely to survive than mice infected with 8325-4 (Pozzi et al., 2012). The 8325-4 HoR strain did not significantly disseminate beyond the surrounding peri-catheter tissue whereas high numbers of the 8235-4 MSSA strain were recovered from the kidneys, liver and spleen (Pozzi et al., 2012). In a murine bacteremia model, mice infected with ΔSCC*mec* and Δ*mecA* mutants of the HA-MRSA strain BH1CC consistently lost more weight during the course of infection and suffered >60% mortality compared to the wild type (Rudkin et al., 2012). These data have led to the hypothesis that MRSA strains have sacrificed virulence for high level antibiotic resistance. Supporting this idea, the infectivity and lethality of MRSA strains was also reduced in guinea pig and murine models of infection compared to MSSA strains (Mizobuchi et al., 1994). Interestingly, MRSA and MSSA isolates were equally virulent in immunocompromised animals (Mizobuchi et al., 1994). Furthermore, a study of 104 patients with *S. aureus* bacteremia found that MSSA bacteremia correlated with significantly higher rates of infective endocarditis than MRSA (Abraham et al., 2004). Similarly, MSSA infections have been associated with a significantly higher illness severity score from invasive disease than MRSA clones (Wehrhahn et al., 2012).

The phenol soluble modulin *mec* (*psm-mec)* locus located adjacent to *mecA* on type II SCC*mec* elements promotes biofilm formation in MRSA strains (Kaito et al., 2011, 2013) and has also been implicated in reduced virulence of MRSA strains in murine models of infection (Kaito et al., 2008, 2011; Queck et al., 2009). The *psm-mec* locus was directly implicated in inhibiting translation of the *agrA* transcript (Kaito et al., 2013). Interestingly carriage of *psm-mec* was only associated with HA-MRSA strains and was absent in the more virulent CA-MRSA isolates and mutation or deletion of the locus from HA-MRSA increased virulence capacity in murine infection models (Kaito et al., 2013). In addition to PSM-*mec*, phenol soluble modulins (PSMs) generally have surfactant qualities and are known to be centrally involved in biofilm structuring and detachment, including dissemination of biofilm-associated infection (Wang et al., 2011; Periasamy et al., 2012). In this context, repression of the Agr system including PSM-*mec* in MRSA is consistent with enhanced biofilm formation.

Oxacillin resistance has pleiotropic effects on *S. aureus*, changing the biofilm phenotype, altering global gene regulation, reducing toxin production and ultimately reducing virulence. However, the importance of MRSA strains as pathogens in healthcare and community setting reflects the high degree of adaptation of *S. aureus* to methicillin resistance and the associated cellular changes. The attenuated virulence associated with high level resistance in HA-MRSA strains, is consistent with their confinement within healthcare settings (Collins et al., 2010; Rudkin et al., 2012), whereas the resistance/virulence equilibrium in CA-MRSA strains has clearly supported their ability in infect otherwise healthy individuals.

### Nucleotide signaling and methicillin resistance

The mechanisms underpinning the relationship between antibiotic resistance and virulence in *S. aureus* remain to be elucidated but studies over recent years implicate nucleotide signaling in these phenotypes. Mutation of the *S. aureus* diadenylate cyclase gene *dacA*, which reduced c-di-AMP levels, resulted in the conversion of a HoR MRSA to a HeR strain (Dengler et al., 2013). Conversely mutations in the *gdpP*-encoded c-di-AMP phosphodiesterase, which resulted in increased c-di-AMP levels were accompanied by HoR to methicillin (Corrigan et al., 2011; Pozzi et al., 2012) or increased tolerance to beta-lactam antibiotics (Griffiths and O'Neill, 2012). In *Bacillus subtilis*, the activity of the GdpP homolog YybT is strongly repressed by the stringent response alarmone ppGpp, which is synthesized by the RelA enzyme (Rao et al., 2010) and Mwangi et al. reported that activation of the stringent response and constitutive ppGpp production was accompanied by homogeneous methicillin resistance (Mwangi et al., 2013). Proteomic analysis has revealed that aminoacyl-tRNA biosynthesis was repressed by subinhibitory concentrations of oxacillin in both MSSA and MRSA (Liu et al., 2014). Reduced levels of aminoacyl-tRNA may trigger the stringent response, which is normally activated by amino acid starvation and specifically the concomitant reduction in charged tRNA levels. A recent genomics analysis of HoR strains derived from a range of clinical HeR isolates identified mutations in 27 genes and 3 intergenic regions, most of which are potentially involved in the stringent response (Dordel et al., 2014). Collectively these data implicate the stringent response in the HoR phenotype and may suggest that ppGpp-mediated repression of GdpP activity leads to reduced c-di-AMP levels and in turn modulates resistance to beta-lactam antibiotics, biofilm and virulence. Identification of c-di-AMP targets may help elucidate the mechanism c-di-AMP-controlled beta-lactam resistance and initial studies to identify c-di-AMP target proteins identified in *S. aureus* and *Listeria monocytogenes* suggest a pleiotrophic role for this nucleotide second messenger (Corrigan et al., 2013; Sureka et al., 2014).

## Concluding thoughts

Biofilm formation is a key virulence factor of staphylococci and distinct mechanisms are employed by MSSA and MRSA for biofilm formation. Clinical MSSA strains predominantly form biofilm dependent on the *icaADBC* operon and PIA production whereas MRSA strains form biofilms independent of PIA (O'Neill et al., 2007). There are important roles for LPXTG-anchored surface proteins, the major autolysin and eDNA during MRSA biofilm formation in the absence of PIA (O'Neill et al., 2008; Houston et al., 2011). Acquisition of methicillin resistance appears to repress polysaccharide-type biofilm production and promote formation of proteinaceous-type biofilms (Pozzi et al., 2012). While the different mechanisms of biofilm formation employed by MSSA and MRSA do not appear to impair the ability of either to colonize implanted biomaterial *in vivo*, the acquisition of methicillin resistance by HA-MRSA is associated with an overall downregulation of virulence gene expression (Pozzi et al., 2012; Rudkin et al., 2012, 2014). Despite this, HA-MRSA strains remain a significant cause of morbidity and mortality for hospitalized patients perhaps reflecting their successful adaptation to a specific niche within the hospital setting where there is a large pool of immunocompromised individuals that often require implanted medical devices and intensive antibiotic treatments (Pozzi et al., 2012). Understanding how MRSA has remained a successful pathogen in the hospital environment, will inform the development of novel therapeutics. Indeed treating MRSA strains with oxacillin may still have therapeutic potential (Rudkin et al., 2014). In CA-MRSA strains, the lower levels of PBP2a expression compared to HA-MRSA is associated with maintenance of high level toxin production. Using oxacillin to increase PBP2a expression can repress Agr activity and secretion of cytolytic toxins, indicating therapeutic potential (Rudkin et al., 2014). Further investigations to assess the global impact of oxacillin treatment on MRSA gene expression are needed but these data illustrate how advances in our understanding of antibiotic resistance and biofilm might be exploited in the development of new strategies to better prevent and treat *S. aureus* infections.

### Conflict of interest statement

The Associate Editor, Joan Goeghegan declares that, despite having collaborated with authors Hannah McCarthy and James P. O'Gara, the review process was handled objectively and no conflict of interest exists. The authors declare that the research was conducted in the absence of any commercial or financial relationships that could be construed as a potential conflict of interest.
